# Study on the Influence of Metal Substrates on Protective Performance of the Coating by EIS

**DOI:** 10.3390/ma17020378

**Published:** 2024-01-12

**Authors:** Jiyun Gong, Han Wei, Pan Hao, Shenghui Li, Xuhui Zhao, Yuming Tang, Yu Zuo

**Affiliations:** Key Laboratory of Carbon Fiber and Functional Polymers, Ministry of Education, Beijing University of Chemical Technology, Beijing 100029, China; 2022210306@buct.edu.cn (J.G.); weihan620@163.com (H.W.); 2022210302@buct.edu.cn (P.H.); 2021210291@buct.edu.cn (S.L.); zuoy@mail.buct.edu.cn (Y.Z.)

**Keywords:** red iron oxide epoxy coating, protection performance, carbon steel, brass, Al alloy, EIS

## Abstract

The degradation process of a red iron oxide epoxy coating on three kinds of metals under a periodic cycling exposure to 3.5 wt% NaCl solution (45 °C 12 h + 25 °C 12 h) was comparatively studied using electrochemical impedance spectroscopy (EIS), Fourier transform infrared spectroscopy (FTIR), scanning electron microscopy (SEM), and X-ray diffraction (XRD) methods. The influence of the metal substrates (carbon steel, brass, and Al alloy) on the protection performance of the coating was analyzed using variations in the electrochemical and chemical parameters. The failure criteria of the coating were discussed. The results show that the coating on the three substrates presents different failure times, with the coating on steel presenting the shortest time and the coating on Al alloy the longest time. The characteristics of metal substrates and their corrosion products influence the coating failure behavior. The corrosion products with loose and hygroscopic properties of steel and brass have promoting effects on the diffusion of water through the coating. The passive film of the Al alloy substrate and the formation of salt film containing Cl^−^ have corrosion-inhibiting effects on the substrate. Evaluation of the coating performance by |Z|_0.01Hz_ should consider the characteristics of the metal substrates.

## 1. Introduction

Organic coatings are one of the most widely used methods for corrosion protection of metal materials. During the service period, corrosive media, including water, oxygen, and ions, penetrate through the micro-pores or other defects in coatings and arrive at the metal surface, leading to the occurrence of corrosion. Different kinds of anti-corrosion pigments, including red iron oxide, mica, talc, and titanium dioxide [1], are incorporated into the polymer matrix to improve the barrier performance. Red iron oxide pigmented epoxy coating is one of the commonly used organic coatings.; it is widely utilized in many fields and can be applied on different metal surfaces because of its good anti-corrosion and anti-permeability performances, good adhesion strength, and relatively low cost [2].

In the study and evaluation of coating performance, electrochemical impedance spectroscopy (EIS) is the most recognized powerful and non-destructive technique [3,4]. By fitting EIS data using equivalent circuit models, some parameters for the coating and the coating/metal interface are obtained [5]. Kouloumbi et al. [6] studied the corrosion resistance and dielectric properties of a red iron oxide epoxy coating by EIS measurements and found that the iron oxides can effectively block the penetration of corrosive media in the coating, thereby improving the shielding effect of the coating on steel substrate. Wang et al. [7] investigated the failure behavior of iron red epoxy coating in marine environment under cyclic wet-dry conditions by EIS and found that the protective performance of the coating on steel can be divided into two stages, the early stage of seawater rapid diffusion and the later stage of the corrosion occurrence on the interface between coating and steel. Ding et al. [8] studied the evolution of the impedance model and the water transport behavior of a solvent-free epoxy coating on Q235 steel by the EIS method. The authors considered that water and ion transports in the coating are important determining factors in the coating failure process to a large extent, and the water transport behavior obeys Fick’s law.

Most studies were carried out at room temperature. It is well known that environmental factors have a big influence on the deterioration of organic coatings, and temperature is a very important influencing factor [9]. Higher temperatures accelerate the diffusion process of corrosive media within the coating. Therefore, the protective property of the coating decreases [10]. Iannarelli et al. [11] found that the cyclic temperature variations in the ballast tank can induce internal stresses in the coating, which has a negative influence on the coating barrier property.

During the coating degradation process, when the electrolyte penetrates the coating/metal interface, the corrosion reactions start to occur on the metal substrate, and the nature or the activity of the metal may have an influence on the corrosion protection characteristics of the coating and the mechanical property of the coating system [2,12,13]. Zhang et al. [14] investigated the water transport behavior of epoxy-coated metals in 3.5% NaCl solution by EIS tests and found that the water diffusion coefficients in the coating are influenced by the type of substrate to some extent. The diffusion rate of water in Al alloy-based epoxy coating is lower than that in the steel-based coating.

In this work, the degradation behavior of a red iron oxide epoxy coating, which was applied on three metal substrates (1020 carbon steel, H62 brass, and 2024-T3 Al alloy), respectively, were studied and compared using EIS, Fourier transform infrared spectroscopy (FTIR), scanning electron microscopy (SEM/EDS), and X-ray diffraction (XRD) tests. The test environment was a 3.5 wt% NaCl solution under a periodic cycling condition (45 °C 12 h + 25 °C 12 h). The purpose of this study is to investigate more clearly the failure mechanism of organic coatings on different metal substrates, the influence of the metals on the corrosion protection characteristics and lifetime of the coatings, and their failure criteria.

## 2. Experimental

### 2.1. Materials and Samples Preparation

The three metal panels were 1020 carbon steel, H62 brass, and 2024-T3 Al alloy, with dimensions of 150 × 75 × 2.5 mm^3^. The panels, which were in the as-received condition, were abraded manually up to 120# abrasive papers to remove the scales and then cleaned with acetone.

A red iron oxide-pigmented epoxy coating was prepared on the three panels, respectively. Firstly, an E-44 epoxy resin (bisphenol A type, produced by Beijing Daishi Zede Technology Co., Ltd., Beijing, China) and an X10 diluent were mixed and stirred for 30 min; then red iron oxide particles were added and mixed intensively. Finally, the T-31 amine curing agent (produced by Beijing Daishi Zede Technology Co., Ltd.) was added and stirred for 15 min to prepare the red iron oxide epoxy coating. The mass ratio of the E-44 epoxy resin to the T-31 amine curing agent was 8.1:1. The coating was coated on the surfaces of the panels by using a spray gun and then cured at 50 °C in the oven for 6 h. The dry film thickness of the coating was measured with a DH-002 coating thickness meter (Shenzhen DONGMEI Measuring Instrument Co., Ltd., Shenzhen, China) at ten random points. The average value was controlled at 80 ± 10 μm.

### 2.2. Experimental Conditions

The coated metal panels were immersed in 3.5% NaCl solution under periodic cycling exposure (45 °C 12 h + 25 °C 12 h) to simulate the marine environment in southern China, which is at a higher temperature in the daytime and a lower temperature at night. The alternating temperature variation was repeated, and the solution was replaced every 48 h.

### 2.3. Measurements

#### 2.3.1. EIS Test

A PARSTAT 2273 instrument (Princeton, NJ, USA) was used to perform EIS tests on the coated metal panels regularly. A traditional three-electrode cell system was used, in which a saturated calomel electrode (SCE) was the reference electrode, a platinum electrode was the counter electrode, and the coated panel was the working electrode. The test solution was a 3.5% NaCl solution at ambient temperature, and the test area of the coated panel was 20 cm^2^. The EIS scans ranged from 10^5^ Hz to 10^−2^ Hz, and the amplitude perturbing signal was 10 mV. There were at least four panels in each condition. The ZsimpWin software (V 3.50, San Diego, CA, USA) was used to fit the EIS data.

A MAS830L digital multimeter (Shenzhen HUAYI Instrument Co., Ltd., Shenzhen, China) was used to measure the open circuit potential (OCP) of the coated metal panels. A CS350 electrochemical workstation (Wuhan CorrTest instruments Co., Ltd., Wuhan, China) was used to monitor the variation in OCP over time for the bared metals of 1020 carbon steel, H62 brass, and 2024-T3 Al alloy in 3.5% NaCl solution up to 48 h, respectively. An SCE electrode was the reference electrode.

#### 2.3.2. FTIR, DSC, SEM, and XRD Tests

The changes in functional groups of the coating were analyzed using a TENSOR27 Fourier-transform infrared spectrometer (FTIR) (BRUKER, Karlsruhe, Germany). The measurements were recorded in the range 500–4000 cm^−1^. The baseline was corrected for the spectra, and the spectra were normalized using the C–H stretching vibration of CH_2_ groups at 2923 cm^−1^.

The glass transition temperature (*T*_g_) of the epoxy coating was determined by a DSC-3 differential scanning calorimeter (Mettler-Toledo, Greifensee, Switzerland) under the conditions of 10 °C/min temperature increase rate from 0 °C to 120 °C. The tests were based on the ASTM D3418-15 standard [15].

The surface morphologies of the coating and the metal substrate were observed by a Quanta 650 scanning electronic microscope (SEM) (FEI, Hillsboro, OR, USA), and the composition analysis was performed by an energy spectrometer (EDS).

The structure of the corrosion products on the substrate underneath the coating was analyzed using the X-ray diffraction (XRD) technique by a 2500VB2+PC X diffractometer (Rigaku, Tokyo, Japan), with CuKα radiation filtered and focused with a Göbel mirror. Before the measurement, the sample was soaked in organic paint remover (25 °C, 24 h), which is mainly composed of dichloromethane and methanol, to remove the coating from the substrate.

## 3. Results and Discussion

### 3.1. EIS Spectra and the Parameters Analysis of Three Metal/Epoxy Coatings

**Figure 1:**
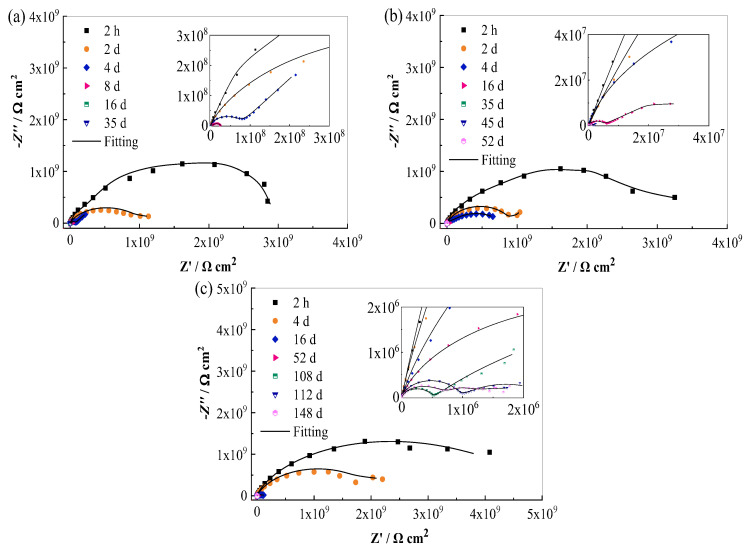
EIS spectra of three kinds of panels: (**a**) steel/coating; (**b**) brass/coating; (**c**) Al alloy/coating.

**Figure 2:**
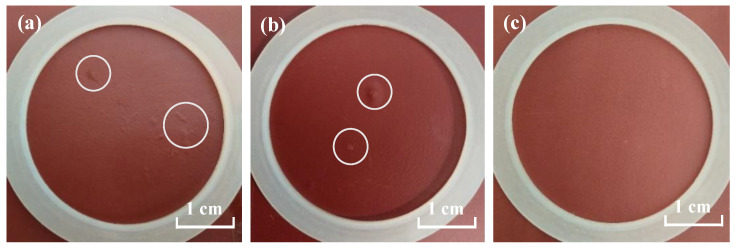
Macro morphologies of the epoxy coatings on: (**a**) steel substrate, 16 d; (**b**) brass substrate, 52 d; (**c**) Al alloy substrate, 148 d.

Figure 1 displays the EIS spectra of the steel/epoxy coating panel, the brass/epoxy coating panel, and the Al alloy/epoxy coating panel under a periodic cycling exposure to 3.5% NaCl solution (45 °C 12 h + 25 °C 12 h). At the beginning (2 h), the Nyquist plots present capacitive arcs with large radii, which indicates the very good barrier property of the coating to the substrates. As the electrolyte continuously diffuses into the coating, the capacitive arc radii decrease gradually. Figure 2 displays the macro morphology using digital photos of the coating panels. It is seen that after 16 d of immersion, several small blisters appear on the surface of the steel/coating panel (Figure 2a), and the area of the blisters gradually grows bigger. Small blisters can be observed on the surface of the brass/coating panel after 52 d of immersion (Figure 2b), while the coating on the Al alloy still presents a flat and intact micro morphology without apparent rust or blisters after 148 d of immersion (Figure 2c). Hence, it can be concluded that there is a big difference in the failure time for the same coating on three different metal substrates, which is in the following order: on the steel substrate < on the brass substrate < on the Al alloy substrate. So, the same epoxy coating can provide much longer protection to the Al alloy.

**Figure 3:**
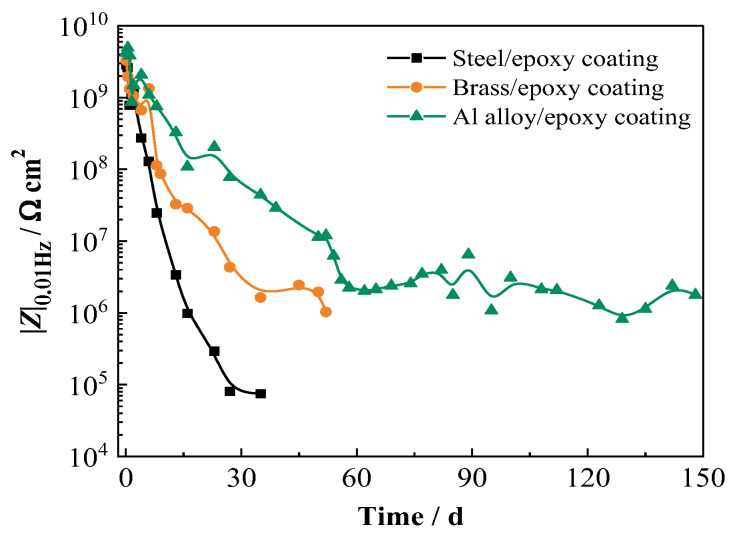
Variations of |Z|_0.01Hz_ with testing time for the epoxy coatings on three metal substrates.

The |Z|_0.01Hz_ variation of the three metal/coating panels was compared, and the results are shown in Figure 3. Initially, the values of |Z|_0.01Hz_ present no big difference, and with extended time, all of them show a rapid decrease. The |Z|_0.01Hz_ of the steel/coating demonstrates the fastest decreasing rate. After 16 d of immersion, it drops to be lower than 1.0 × 10^6^ Ω cm^2^ (about 9.8 × 10^5^ Ω cm^2^), and continuous to 7.5 × 10^4^ Ω cm^2^ after 35 d test. The decreasing rate of |Z|_0.01Hz_ for brass/coating is relatively slower. After 35 d of immersion, the |Z|_0.01Hz_ is 1.6 × 10^6^ Ω cm^2^ and decreases to 1.0 × 10^6^ Ω cm^2^ at 52 d. For the Al alloy/coating, after 60 d of immersion, the value of |Z|_0.01Hz_ is about 2.2 × 10^6^ Ω cm^2^ and then tends to be steady, and until 148 d, it is still around 1.8 × 10^6^ Ω cm^2^. The low-frequency impedance (|Z|_0.01Hz_) of an organic coating system can be used as a general indicator of its performance. The coating with |Z|_0.01Hz_ over 10^9^ Ω cm^2^ provides excellent corrosion protection to the substrate, while that lower than 10^6^ Ω cm^2^ provides poor corrosion protection [16,17]. However, this is typically for coated steel systems [18]. For the coated brass and Al alloy systems, no related thresholds have been reported yet. The primary results in Figure 2 and Figure 3 indicate that the threshold |Z|_0.01Hz_ for the coated Al alloy should be different from those for the coated carbon steel and brass. For the coated steel and brass, when the value of |Z|_0.01Hz_ is close to 1.0 × 10^6^ Ω cm^2^, the coating provides poor corrosion protection manifested by the blisters observed. However, when the |Z|_0.01Hz_ value of the coated Al alloy is around 1.0 × 10^6^ Ω cm^2^, the coating still stays in good condition for a long time without any blisters or rust, which indicates that the failure threshold of |Z|_0.01Hz_ for the coated Al alloy might be lower than 1.0 × 10^6^ Ω cm^2^. Table 1 shows the |Z|_0.01Hz_ values of three coated metal panels with testing time and the corresponding protection performance of the epoxy coating.

**Figure 4:**
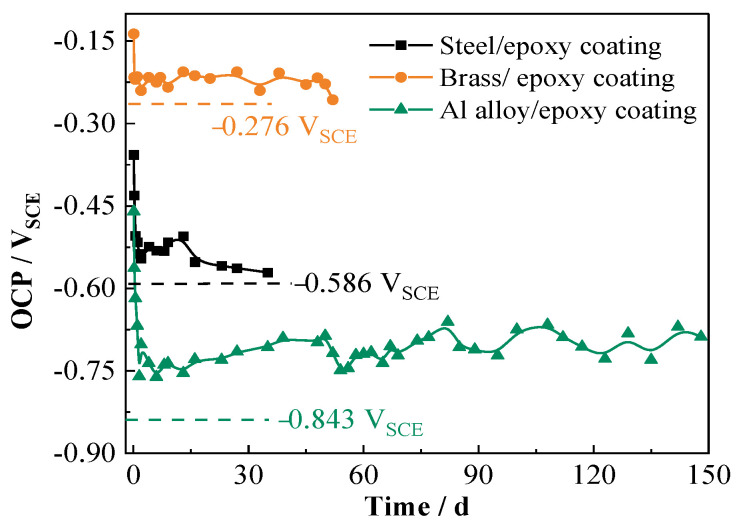
Variations in OCP over time for the three coated metal panels.

Figure 4 shows the variations in OCP with testing time for the three coated metal panels, in which the OCP of each bare metal in 3.5% NaCl solution is also presented with a dotted line, which is −0.276 V_SCE_, −0.586 V_SCE_, and −0.843 V_SCE_ for the brass, steel, and Al alloy, respectively. It is seen that the OCP values of all the coated panels decrease quickly at the initial stage because of water uptake of the coating and then gradually tend to be stable. In the relatively stable stages, the OCP of the coated brass is at approximately −0.22 V_SCE_, and the one for the coated steel is around −0.54 V_SCE_, which both are a little higher than the OCP of the corresponding bare metals. These indicate that the solution has already reached the substrate and causes the corrosion [19,20]. The OCP value for the coated Al alloy in the 148 d testing period is about −0.74 V_SCE_, which is apparently higher than that of the bare Al alloy. This demonstrates that the epoxy coating exhibits a more long-lasting barrier effect to the Al alloy substrate. Therefore, OCP can be used as a supplementary measure reflecting the stability of the coating and, therefore, its barrier property.

**Figure 5:**
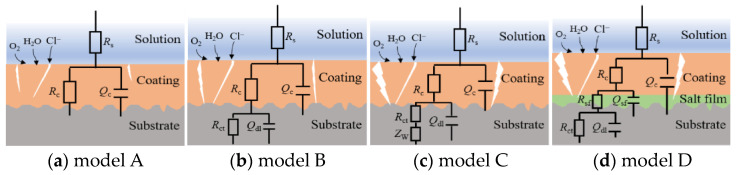
Equivalent circuit models used to fit the EIS data.

The equivalent circuit models in Figure 5 were used to fit and analyze the impedance data. Figure 5a shows the model for the initial immersion stage, in which *R*_s_ is the solution resistance, *Q*_c_ is the constant phase element (CPE) related to the non-ideal capacitance behavior of the coating system, and *R*_c_ is the coating resistance [17]. After immersion for 3–4 d under the test condition, the electrolyte reaches the coating/metal interface, and corrosion occurs on the substrate; the model in Figure 5b was used, in which *Q*_dl_ and *R*_ct_ represent the double layer capacitance and the charge transfer resistance which are related to the electrochemical corrosion reactions. After 16 d, 45 d, and 82 d of immersion for the coated steel, brass, and Al alloy panels, respectively, the model in Figure 5c was used, in which a Warburg impedance (*Z*_w_) was added, corresponding to the diffusion of corrosion products through the micro-pores in the coating [19]. For the Al alloy/coating system, after the Cl^−^ ions penetrate through the coating and reach the alloy/coating interface, they will participate in the chemical reactions with aluminum hydroxide corrosion products to form a salt film on the alloy surface, which results in the changes in the impedance spectra. So, Model D in Figure 5d was used to fit the data after immersion for 112 d, in which *Q*_sf_ is salt film capacitance and *R*_sf_ is salt film resistance [14,21].

**Figure 6:**
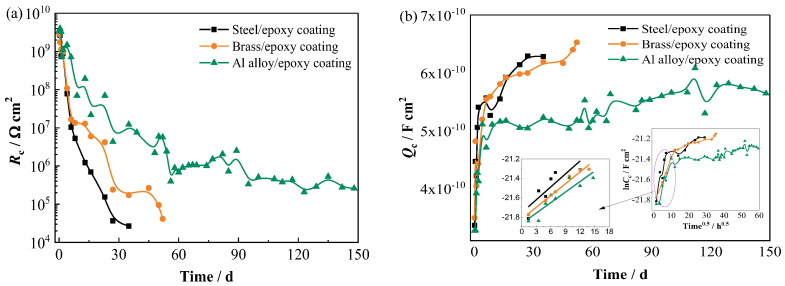
Variation in (**a**) *R*_c_ and (**b**) *Q*_c_ over time for three coated metal panels.

The changes in *Q*_c_ and *R*_c_ for the three coated panels as a function of time are presented in Figure 6a,b. At the initial stage, due to the rapid water uptake of the coating, the *R*_c_ decreases quickly, and the *Q*_c_ increases rapidly for all the coated panels; thus, the values in the first several days present no significant difference. With extended time, the *R*_c_ and *Q*_c_ of the coated Al alloy panel gradually stabilize and maintain the lowest capacitance and highest resistance values for a long period, manifesting the lowest water absorption of the coating on the Al alloy substrate. Comparing the data of the coatings on the steel and brass substrates, the *R*_c_ is slightly lower, and the *Q*_c_ is slightly higher for the coated steel panel at the same time, which indicates a slightly more rapid water uptake speed of the coating on the steel.

The coating capacitance *C*_c_ can be calculated by *Q*_c_ according to Equation (1), in which *R*_s_, *R*_c_, and *n* are fitting elements for the equivalent circuit shown in Figure 5 [22]. Then, the capacitance–time curve (ln*C*_c_ − *t*^0.5^) for the three metal/coating panels is obtained, and the enlarged parts, which contain a linear relationship at the initial immersion stage, are presented in Figure 6b. This confirms that the diffusive behavior of water in the coating follows Fick’s law in the initial immersion stage, which is 0–6 d, 0–8 d, and 0–9 d for the coating on the steel, brass, and Al alloy substrates, respectively. Therefore, the water diffusion coefficient (*D*) of the coating can be obtained from the time dependence of the coating capacitance in the case of Fickian diffusion by using Equation (2) [14,21]. Where *C*_0_ is the coating capacitance at initial time (*t* = 0), *C*_t_ is the coating capacitance at time t, *C*_∞_ is the coating capacitance at saturation, and *L* is the coating thickness. The calculated water diffusion coefficient for the coating on the steel, brass, and Al alloy substrate is 4.53 × 10^−9^ cm^2^/s, 4.38 × 10^−9^ cm^2^/s, and 3.67 × 10^−9^ cm^2^/s, respectively. The values are consistent with the literature, in which the same order of magnitude of *D* in epoxy coating (2.44 × 10^−9^ cm^2^/s, 2.10 × 10^−9^ cm^2^/s) was reported [21,23]. It is noted that the obtained *D* of the coating on the three metal substrates is different among them, as the value for the coating on the Al alloy substrate is the smallest one, and the values for the coatings on brass and steel are relatively larger and close, and the one on the brass substrate is slightly smaller than that on the steel. This probably is due to the hygroscopicity of the reaction products on the metals. After water, oxygen, and corrosive ions reach the steel/coating interface and brass/coating interface, hydroscopic products are formed, which will attract more solution penetrating through the coating from the outside to the anodic corroded sites under the driving force of osmotic pressure [24,25], thereby leading to higher water diffusion coefficients.

*C*_c_ = *Q*_c_^1/n^ (*R*_s_^−1^ + *R*_c_^−1^)^(n−1)/n^ (1)

(2) $$\frac{lnC_{t}-lnC_{0}}{lnC_{\infty }-lnC_{0}}=\frac{2\sqrt{t}}{L\sqrt{\pi }}\sqrt{D}$$

**Figure 7:**
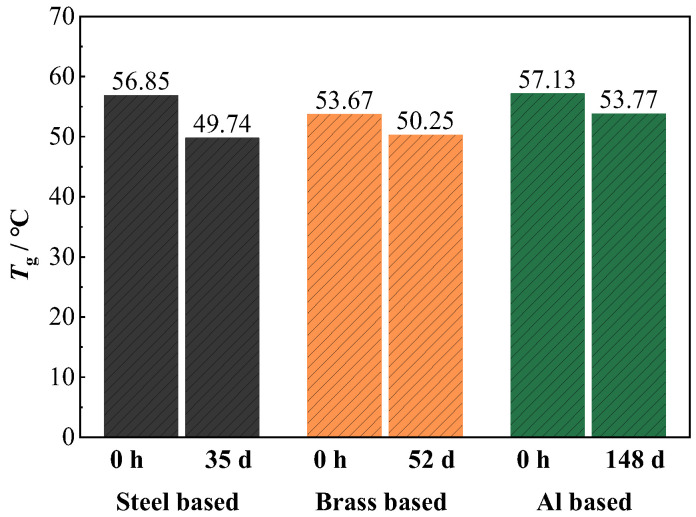
*T*_g_ results of the coating on different metal substrates.

Figure 7 displays the glass transition temperature (*T*_g_) results of the coatings on different metals determined by DSC analysis. Before immersion, the *T*_g_ value of the coating on the steel, brass, and Al alloy is 56.85 °C, 53.67 °C, and 57.13 °C, respectively. After immersion under the periodic cycling exposure condition for a period of time, the *T*_g_ values of the coatings on the three metals all decrease. For the coating on the steel, the value of *T*_g_ decreases to 49.74 °C after 35 d of immersion. For the coating on the brass substrate, it decreases to 50.25 °C after 52 d of immersion. Concurrently, the *T*_g_ value of the epoxy coating on the Al alloy substrate decreases to 53.77 °C even after 148 d of immersion with the smallest decreasing amplitude. As we know, *T*_g_ is an important parameter for polymers and is closely related to their density and the cross-linking degree. Usually, the higher the cross-linking, the higher the *T*_g_ value. So, *T*_g_ is related to the barrier property of the polymer coatings against water, O_2_, and ions [14,26]. After immersion, the *T*_g_ value of the coating decreases because the penetrated water may plasticize the cross-linked polymers and disrupt inter-chain hydrogen bonds [27]. The higher *T*_g_ of the coating on the Al alloy demonstrates that the coating possesses a more compact structure, and its chemical properties are less likely to deteriorate due to water penetration. This is in good agreement with the result of the water diffusion coefficient. In the literature, it was also reported that the decrease in *T*_g_ of coatings on Al alloy is smaller than that on mild steel after a period of immersion, which is consistent with our research [14,27].

**Figure 8:**
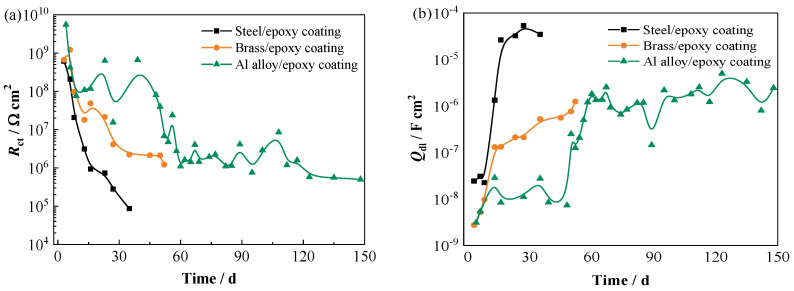

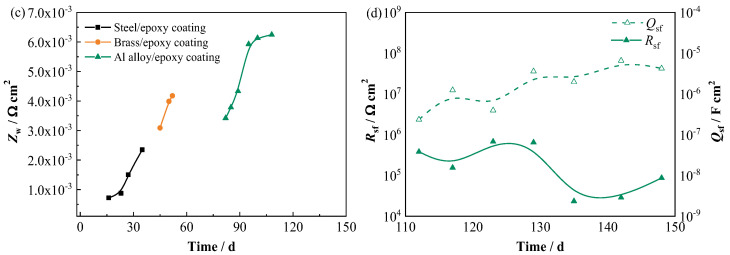
Variations in (**a**) *R*_ct_, (**b**) *Q*_dl_, (**c**) *Z*_w_, and (**d**) *R*_sf_ over time for three coated panels.

*R*_ct_ is often used to evaluate the electron transfer activity at the interface between the coating and the metal substrate. The higher the *R*_ct_ value, the lower the corrosion rate [28]. The variations of *R*_ct_ for three different panels are presented in Figure 8a. It can be seen that all the *R*_ct_ values begin to appear at 3–4 d. The *R*_ct_ on both steel and brass substrates shows a continuous downward trend during the testing time, demonstrating the increase in the electron transfer activity at the coating/metal interface [29]. In contrast, the *R*_ct_ on the Al alloy substrate is the highest and presents a relatively slower downward trend and tends to be steady after the 60 d test, which might be related to the passive film on the Al alloy substrate that can inhibit corrosion reaction. The steady curve of the Al alloy-based panel at the middle and later stages probably has a relationship with the formation of salt film incorporated with chloride ions on the substrate [14,27]. Both the high values of *R*_c_ and *R*_ct_ demonstrate that the coating on the Al alloy substrate has very good barrier performance against water, which can protect the substrate from serious corrosion.

Generally, *Q*_dl_ can reflect the electrochemical reaction area on the metal [10,23]. The evolution processes of *Q*_dl_ for the three panels are shown in Figure 8b. It is seen that in the same time range, the coated Al alloy presents the lowest *Q*_dl_ value, indicating a much lower electrochemical reaction area on the Al alloy substrate. The coated steel presents the highest *Q*_dl_ value, implying the highest electrochemical activity on the steel substrate. Figure 8c shows the variation in Warburg impedance (*Z*_w_) over time. It can be seen that the presence of *Z*_w_ for the steel-based, brass-based, and Al alloy-based panels is 16 d, 45 d, and 82 d, respectively. As we know, with extended time, more corrosion products accumulate on the metal substrate surface, and the diffusion of the products from the metal surface towards the coating may be impeded by the coating. Hence, the diffusion process may become a control procedure in the Faradaic processes [14,27]. Because corrosion products are formed on the metal surface, a thermodynamic water activity difference is established between the corroded sites underneath the coating and the solution exposed. Therefore, an osmotic pressure gradient is established, which will drive water migrating through the coating, and meanwhile, the blisters are formed [25]. Hence, the water diffusion coefficient and the water uptake of the coatings on the steel substrate and the brass substrate are higher than that on the Al alloy. After 112 d of test in the Nyquist spectrum for the coated Al alloy panel, the low-frequency tail disappears and is displaced by another loop (Figure 1c) [21], which indicates that the soluble corrosion products originally accumulated at Al alloy/coating interface are possibly consumed because, after long-time immersion in NaCl solution, the Cl^−^ ions reach the Al alloy substrate and participate in the chemical reactions with corrosion products to form a salt film [25,27]. As can be seen from Figure 8d, with extended time, the salt film resistance *R*_sf_ decreases slowly, and the salt film capacitance *Q*_sf_ increases. The formation of the salt film might be an important reason that the red iron oxide epoxy coating on the Al alloy substrate can provide longer lasting protection in the NaCl solution. So, metal substrates have certain effects on the corrosion-resistant performance of the coating and, therefore, influence the service lifetime of the coating.

### 3.2. FTIR Results of the Red Iron Oxide Epoxy Coating

**Figure 9:**
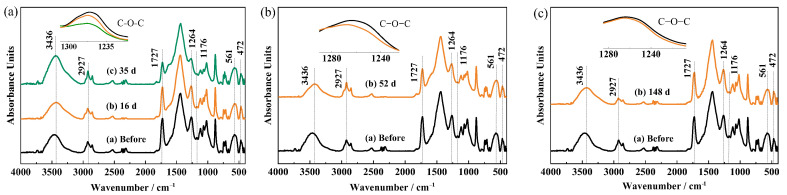
FTIR spectra of the coating on three metal substrates (**a**) steel, (**b**) brass, (**c**) Al alloy.

Figure 9 presents the infrared spectra of the red iron oxide epoxy coating on the three metal substrates before and after the test. In the spectra, the broad band with a peak at 3436 cm^−1^ can be attributed to the stretching vibration of –OH groups (hydroxyl) [30,31], the peak at 2927 cm^−1^ is due to the stretching vibration of –CH_2_ (aliphatic hydrocarbon) bands [23,31], the peak at 1727 cm^−1^ is due to C=O (carbonyl) stretching of esters [30,32], the peak at 1264 cm^−1^ represents the C–O–C (ether) bands [33,34], the peak at 1176 cm^−1^ represents C–N bond of the amine hardener [33,34], and peaks at about 561 cm^−1^ and 472 cm^−1^ correspond to Fe–O vibration modes [2]. It can be seen from the spectra of the coating on steel (Figure 9a) that after 16 d of immersion, the peak intensity of 1264 cm^−1^ decreases slightly, and after 35 d, the intensities of 1727 cm^−1^, 1264 cm^−1^, and 1176 cm^−1^ all decrease apparently. The spectra of epoxy coating on brass (Figure 9b) show that after 52 d, the intensities of 1727 cm^−1^ and 1264 cm^−1^ also slightly decrease. These changes demonstrate that after immersion for a period of time, some chemical bonds, especially carbonyl and ether groups, in the epoxy coating resin are broken, which will cause the porosity to increase and more channels to form for the solution permeation through the coating [35]. For the Al alloy/coating (Figure 9c), after 148 d immersion, the FTIR spectra present no big change, indicating no apparent degradation occurring. It was reported that the absorbed water in the coating could plasticize the polymer resin, and some of the water will react with the polymer through a hydrolysis reaction, causing the epoxy backbone chains or crosslink chains to experience scission [36,37]. So, the interaction between water and epoxy resin plays an important role in the degradation of the epoxy coating [38]. The above EIS results show that the water uptake in the coating on the Al alloy is relatively lower, so there might be less chain scission induced by the hydrolysis reaction. Therefore, even after 148 d of immersion, the change in the spectra of the coating on the Al alloy is still not obvious.

### 3.3. Surface Morphology and the Corrosion Products Analysis

**Figure 10:**
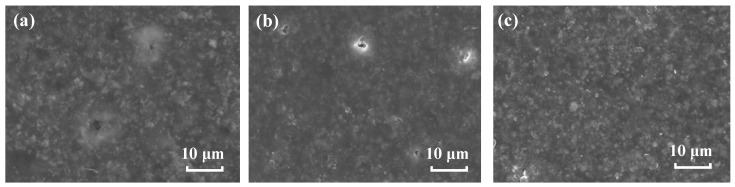
SEM surface images of the coating on (**a**) steel, 35 d (**b**) brass, 52 d (**c**) Al alloy, 148 d.

Figure 10 shows the surface morphology of the coating on three metals after immersion. It can be seen from Figure 10a,b that after 35 d and 52 d of immersion, respectively, some micro-pores and defects occur on the coating surface on the steel and brass substrates. These might arise from the water uptake of the coating, which can induce swelling, hydrolysis, and cracking in the coating [39,40]. The swelling can produce net stresses, which might open up some micro defects for the water and ions penetrating and permit more water to pass [12], therefore accelerating the deterioration of the coating and losing the protection performance of the substrate. The coating on the Al alloy substrate presents relatively intact morphology with no obvious micro-pores and defects after 148 d of immersion (Figure 10c). This is consistent with the relatively lower water absorption result by EIS analysis for the coating on the Al alloy.

**Figure 11:**
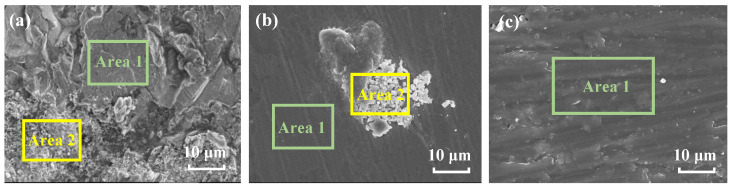
SEM morphology of the substrates: (**a**) steel after 35 d (**b**) brass after 52 d (**c**) Al alloy after 148 d.

Figure 11 shows the surface morphology of the three metal substrates after the coating was removed. It can be seen in Figure 11a that after 35 d of immersion, the surface of the steel is partly covered with some corrosion products; here, Area 2 is the corroded area, and Area 1 is the area with no products. In Figure 11b, after 52 d of immersion, corrosion products are also observed on the surface of brass (Area 2), and the scratches caused by polishing with sandpapers can be observed on the area with no corrosion signs (Area 1). After 148 d of immersion, the surface of Al alloy presents a flat and integrated morphology (Figure 11c). This is probably owing to the restively lower electrochemical reaction activity of the Al alloy and may also have a relationship with the formation of the salt film containing Cl^−^ ions, which inhibits the corrosion process and modifies the surface morphology of the alloy substrate [21]. EDS test was performed on the surface of three metals, and the results are shown in Table 2. The elements, including C, O, Cl, and Fe, are detected on the steel surface, in which the contents of O and Cl in Area 2 are higher than those in Area 1. This indicates that the surface of Area 2 suffers more severe corrosion, and the corrosion products are iron oxides and compounds containing chlorine. On the brass substrate, the main elements detected are C, Cu, Zn, O, and Cl, and similarly, the contents of O and Cl in Area 2 are much higher than those in Area 1. This means that the corrosion products on brass might be the oxides containing copper and zinc with some chloride. The amounts of O and Cl elements on the Al alloy substrate are much lower than those detected on steel and brass. This implies that the Al alloy substrate suffers relatively less corrosion, which is in good agreement with the above results of EIS and FTIR.

**Figure 12:**
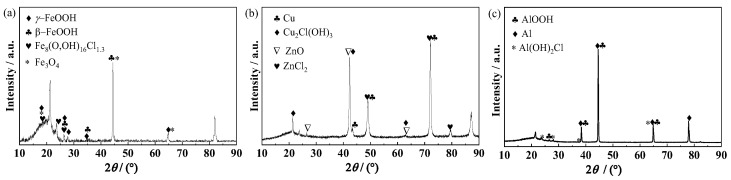
XRD results of corrosion products on the metal substrates: (**a**) steel after 35 d, (**b**) brass after 52 d, (**c**) Al alloy after 148 d.

XRD measurement was performed to analyze the corrosion product compositions and the spectra are shown in Figure 12. In the result on the steel substrate (Figure 12a), It is seen that the corrosion products on the steel mainly consist of *β*-FeOOH, *γ*-FeOOH, Fe_8_(OOH)_16_Cl_1.3_ and Fe_3_O_4_ [41,42,43]. It was reported that in the initial stage of steel corrosion, the reaction product is Fe(OH)_2_, which is a loose and porous corrosion product [41]. As corrosion proceeds, Fe(OH)_2_ is gradually dissolved in the thin liquid film at the interface between the steel and the coating [42], forming a high-concentration salt solution and building up an osmotic pressure between the anodic active sites with the solution outside the coating, which can promote more water diffusing into the coating [24,25,44]. After sufficient oxygen has accumulated at the interface, the Fe(OH)_2_ can be oxidized to FeOOH [41,42], and part of FeOOH may transform into more stable Fe_3_O_4_ [42,43]. The reduction of FeOOH can promote the cathodic reaction (2H_2_O + O_2_ + 4e^−^ → 4OH^−^) [43]. Therefore, more water is needed for the cathodic reaction, and the water transport channel increases [9]. All of these can accelerate the corrosion of the steel substrate. Figure 12b is the XRD result of the corrosion products on the brass substrate. It is identified that the main phases of the corrosion products are ZnO, ZnCl_2_, and Cu_2_Cl(OH)_3_ [45,46]. It was reported that ZnCl_2_ is a hydrosoluble substance, and Cu_2_Cl(OH)_3_ is loose and easy to deliquesce [46], which easily leads to osmotic pressure and, therefore, a further increase in water uptake in the coating/brass system [9,24,25]. Figure 12c is the result of the analysis of the corrosion products on the Al alloy substrate. It is seen that the strongest diffraction peaks are the characteristic peaks for the Al substrate. In addition, the characteristic peaks of AlOOH (Al_2_O_3_·H_2_O) are identified, demonstrating the existence of the insoluble AlOOH passive film on the substrate, which has inhibiting effects on the corrosion of Al alloy [47]. The Al(OH)_2_Cl compound is also detected. The possible reason is that the arriving Cl^−^ ions participate in the chemical reactions with aluminum ions to form Al(OH)_2_Cl, which significantly impedes the corrosion process of the Al alloy substrate [21,26]. The characterization result is consistent with the above assumption of the formation of Cl^−^ participating salt film in an equivalent electrical circuit (Model D in Figure 5d). The Al alloy and its corrosion products have no obvious promoting effects on the water diffusion in the epoxy coating.

## 4. Conclusions

(1) The red iron oxide epoxy coating on three different metal substrates presents different failure times in a periodic cycling exposure to 3.5 wt% NaCl solution (45 °C 12 h + 25 °C 12 h), which is in the following order: the coating on steel < the coating on brass < the coating on Al alloy. The anti-corrosion property and the service lifetime of the coating have a relationship with the metal substrates.
(2) The characteristics of the metal substrates and their corrosion products have a significant influence on the water uptake and, thereby, the degradation process of the coating. The corrosion products on steel and brass contain hygroscopic compounds, which have a promoting effect on water intrusion and accelerate the corrosion process of the substrates. The passivity of Al alloy and the formation of Cl^−^ containing salt film can inhibit the corrosion of substrate and have no significant promoting effect on the water diffusion in the coating.
(3) The failure threshold |Z|_0.01Hz_ for the coating on Al alloy should be different from that on carbon steel and brass. For the coating on steel and brass, the value of |Z|_0.01Hz_ is close to 1.0 × 10^6^ Ω cm^2^, and the value of |Z|_0.01Hz_ for the coating on Al alloy is suggested to be lower than 1.0 × 10^6^ Ω cm^2^.

## Figures and Tables

**Table 1:** |Z|_0.01Hz_ values of three coated panels and the protection performance of the epoxy coating.

Time	Steel/Epoxy Coating		Brass/Epoxy Coating		Al Alloy/Epoxy Coating	
	|*Z*|_0.01Hz_/Ω cm^2^	Protection Performance	|*Z*|_0.01Hz_/Ω cm^2^	Protection Performance	|*Z*|_0.01Hz_/Ω cm^2^	Protection Performance
2 h	2.8 × 10^9^	Excellent	3.3 × 10^9^	Excellent	4.2 × 10^9^	Excellent
4 d	2.7 × 10^8^	Very good	6.7 × 10^8^	Very good	2.1 × 10^9^	Excellent
16 d	9.8 × 10^5^	Poor	2.8 × 10^7^	Good	1.1 × 10^8^	Very good
35 d	7.5 × 10^4^	Failure	1.6 × 10^6^	Poor	4.4 × 10^7^	Good
52 d			1.0 × 10^6^	Failure	1.2 × 10^7^	Good
148 d					1.8 × 10^6^	Good

**Table 2:** EDS results of the three substrate surfaces after immersion.

Element (wt%)	C	O	Cl	Fe	Cu	Zn	Al	Mg
Steel (Area1)	17.56	6.54	0.13	75.78	—	—	—	—
Steel (Area2)	15.06	16.78	0.40	67.76				
Brass (Area1)	21.59	6.18	0.34	—	44.80	27.09	—	—
Brass (Area2)	31.14	20.13	6.32	—	9.96	32.45	—	—
Al alloy (Area1)	24.62	3.04	0.05	—	—	—	69.64	2.65

## Data Availability

Data are contained within the article.
